# Stock Price Movement Prediction Using Sentiment Analysis and CandleStick Chart Representation

**DOI:** 10.3390/s21237957

**Published:** 2021-11-29

**Authors:** Trang-Thi Ho, Yennun Huang

**Affiliations:** Research Center for Information Technology Innovation, Academia Sinica, Taipei 10607, Taiwan; yennunhuang@citi.sinica.edu.tw

**Keywords:** stock market, sentiment analysis, candlestick chart, convolution neural network

## Abstract

Determining the price movement of stocks is a challenging problem to solve because of factors such as industry performance, economic variables, investor sentiment, company news, company performance, and social media sentiment. People can predict the price movement of stocks by applying machine learning algorithms on information contained in historical data, stock candlestick-chart data, and social-media data. However, it is hard to predict stock movement based on a single classifier. In this study, we proposed a multichannel collaborative network by incorporating candlestick-chart and social-media data for stock trend predictions. We first extracted the social media sentiment features using the Natural Language Toolkit and sentiment analysis data from Twitter. We then transformed the stock’s historical time series data into a candlestick chart to elucidate patterns in the stock’s movement. Finally, we integrated the stock’s sentiment features and its candlestick chart to predict the stock price movement over 4-, 6-, 8-, and 10-day time periods. Our collaborative network consisted of two branches: the first branch contained a one-dimensional convolutional neural network (CNN) performing sentiment classification. The second branch included a two-dimensional (2D) CNN performing image classifications based on 2D candlestick chart data. We evaluated our model for five high-demand stocks (Apple, Tesla, IBM, Amazon, and Google) and determined that our collaborative network achieved promising results and compared favorably against single-network models using either sentiment data or candlestick charts alone. The proposed method obtained the most favorable performance with 75.38% accuracy for Apple stock. We also found that the stock price prediction achieved more favorable performance over longer periods of time compared with shorter periods of time.

## 1. Introduction

The stock market is a major topic in modern life. Investors can easily purchase new stocks and gain significant profits from dividends provided in the company’s bonus program for shareholders. Investors can also trade their own stocks with the other traders in the stock market via stock brokerages and electronic trading platforms. Stock traders wish to buy stocks with values that are expected to increase and sell stocks with values that are expected to decrease. Therefore, stock traders must be able to predict the trends of a stock’s behavior before making a trading decision to buy or sell a stock. The more accurate their prediction about the behavior of a stock is, the more profit they earn. Therefore, developing an automatic algorithm that can accurately predict market trends to help traders maximize profit is essential. However, determining stock market trends is a challenging problem because of factors such as industry performance, company news, company performance, investor sentiment, economic variables, and social media sentiment.

According to Fama’s efficient market hypothesis [1], investment in the stock market involves risk, and the possibility of investors gaining an advantage by buying underrated stocks or selling stocks for an inflated price does not exist. To avoid high-risk stocks and secure higher profits, a stock trader has only one means of evaluating a company’s performance before purchasing its stock. With the development of technological advances, deep learning—especially convolutional neural networks (CNNs)—has exhibited favorable performance in a range of research fields [2,3,4,5,6,7,8,9,10,11,12,13,14,15]. Many researchers have applied deep learning to the question of stock market prediction. There are several approaches for stock market prediction, such as analyzing indicators of historical time series data [16,17,18,19,20], or using candlestick chart converted from historical data [21,22,23,24,25,26], or analyzing the social media [27,28,29,30,31,32], or analyzing the financial news [33,34,35,36]. However, using a single classifier may not achieve maximum performance compared with using combined classifiers. Different classifiers can be combined using ensemble methods in machine learning to improve the prediction accuracy of individual classifiers. In this study, we proposed a novel framework for stock market prediction that incorporates both candlestick charts and sentiment analysis of social-media data as internal and external factors. To our knowledge, this is the first study to integrate candlestick charts and sentiment data from social media for the purpose of stock prediction.

Because social-media data are text data from tweets and candlestick chart data comprise color images, the information requires extraction and processing before being incorporated into a collaborative network. For social-media data, the Natural Language Toolkit (NLTK) was used for sentiment analysis of the content of tweets. We removed spam tweets and irrelevant information to help our model achieve accurate sentiment score features. The candlestick chart data were generated by mpl_finance module [37] by using four features (open, high, low, and close) of historical time series data. Data from candlestick charts were normalized before being input into a CNN for extraction of internal features. A multibranch network was designed to incorporate features from social-media and candlestick-chart data. The output of these branches was concatenated in a dense layer. The last layer of the multibranch network contained two neurons to classify the stock trend as increasing or decreasing in the near future.

In summary, this study makes the following contributions:

- We employed a one-dimensional (1D) CNN (1D-CNN) that performed more favorably than traditional machine learning and long short-term memory models for stock trend prediction based on social-media data.
- We proposed a novel collaborative deep learning network for stock-price-movement prediction. The main features were incorporated internal (candlestick chart) and external (social media sentiment) stock features that helped to improve the accuracy of the prediction of stock price movement.
- We formulated a new accuracy-level metric for comparing the accuracy ranges between classification models for stock trend prediction.
- We observed that price movement prediction for a “high-demand stock” achieved more favorable performance in the long term.

In the remainder of this article, we first review related literature in Section 2. We then explain our methodology in Section 3. Section 4 presents the experimental evaluations, and we finally conclude the article in Section 5.

## 2. Related Works

Many researchers have developed various prediction models for stock market prediction to help traders make the correct decision on their stocks. In 1990, Schneburg [38] conducted a study on the German stock market. A machine learning architecture with back-propagation was built based on historical time-series data. In 2007, Khoa [39] proposed a simply recurrent neural network for stock prediction and concluded that this simply recurrent neural network achieves better results compared to back-propagation neural networks due to its “capturing capabilities.” After that, Ou and Wang [20] utilized the Hong Kong stock market to build a stock market prediction model with ten machine learning algorithms. They demonstrated that SVM and LS-SVM achieve better performance than the other methods. In 2016, Chen proposed planar feature representation methods and deep convolutional neural networks to improve the algorithmic trading framework [40].

In the last decade, sentiment analysis has carried great importance because of the huge amount of textual data on news and social media platforms. There is a significant amount of research on mining opinions of users for different application areas [41,42,43,44,45]. Ali performed sentiment analysis to detect transportation entities in the large corpus [42] and to detect traffic accidents to reduce serious injuries [43]. Basiri proposed a sentiment analysis method on the Twitter dataset using the attention-based bidirectional CNN-RNN deep model [44]. Li [45] proposed a bidirectional emotional recurrent unit for exploring the emotion in conversation. Using the same trend for stock prediction, some researchers have started to utilize sentiment analysis to analyze the stock market’s movements. In 2011, J. Bollen proposed a sentiment analysis method to predict the movement of the Dow Jones Industrial Average (DJIA) stock market based on the famous microblogging site Twitter. Khatri and Srivastava [30] explored the relationship between text sentiment from tweets, comments on the Stock Twist website, and the stock price of Facebook, Apple, Google, Oracle, and Microsoft stocks from Yahoo Finance. In 2017, Urolagin used an SVM classifier to perform sentiment classification and predict stock market status based on stock price data from Yahoo Finance and social-media data from Twitter [27]. He explored an association between tweet features and the stock prices of a company. Not long after that, Chakraborty proposed a stock-market-movement prediction model based on tweets’ sentiment of Appple Inc stock [28]. The SVM classifier was used for sentiment classification, while the stock movement prediction model was trained by applying the boosted regression tree. In 2020, Khan [46] utilized algorithms on social media and financial news data to discover the impact of both data on stock movement.

Many researchers have found different methods to approach stock data; they transformed the historical time series data into other forms to learn more patterns inside the stock movement. Candlestick charts have been used to visualize the daily price and stock market movement. In 2013, Prado utilized sixteen candlestick patterns to forecast stock movement for Brazilian stocks [26]. Tsai combined candlestick charts with seven different wavelet-based textures to predict stock movement [22]. In 2017, Hu employed a convolutional encoder to learn the candlestick chart patterns to build a decision-making system for the stock market. Our previous work [21] proposed a deep convolutional neural network for stock market prediction based on candlestick charts for two different stock markets (Taiwan50 and Indo10). In 2020, Birogul [25] employed a real-time object detection system (YOLO) to recognize buy–sell objects inside 2D candlestick charts; from these buy–sell objects, the trader can make their decision on the stock. Not long after that, Hung [24] proposed a deep predictor framework for price movement based on candlestick charts. He explored a CNN-autoencoder to acquire the best sub-chart representation and applied recurrent neural networks to predict the stock price movement.

Unlike existing methods, we did not solely rely on sentiment analysis of social media posts or explore patterns in the candlestick charts. In this study, we proposed a collaborative network incorporating candlestick charts and social-media data for stock movement prediction. To our knowledge, this is the first study to use both candlestick charts and social sentiment data to predict stock prices. Using both types of data is more effective in predicting stock trends because both types of data can change and affect movements in a stock’s price and in traders’ decisions.

## 3. Approach

**Figure 1:**
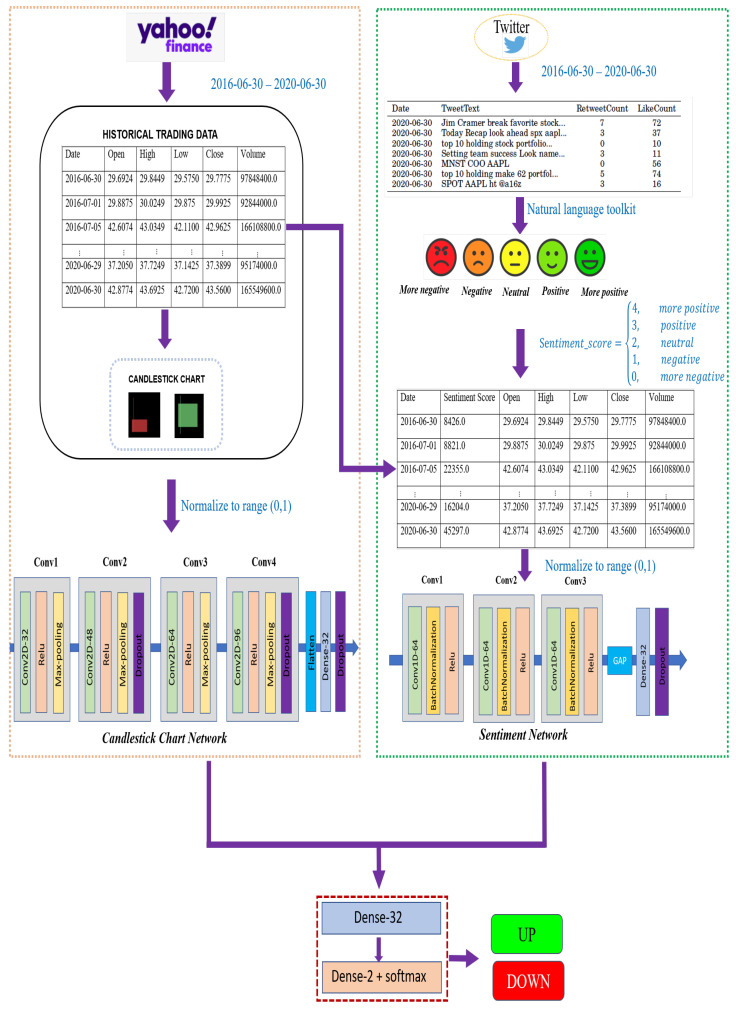
Architecture of the multibranch network for stock trend prediction.

In this section, we present a joint multichannel framework for stock movement prediction. This section is organized as follows: first, we introduce the sentiment analysis for stock trend prediction in Section 3.1. We then describe the candlestick chart generation and its branch network for stock price movement in Section 3.2. Finally, we present a collaborative network incorporating all the features from sentiment analysis and candlestick charts that helps to improve prediction performance in Section 3.3. Section 3.4 introduces performance measures utilized to evaluate our classification model performance. Figure 1 exhibits the architecture of our stock prediction framework.

### 3.1. Sentiment Analysis for Stock Trend Prediction

#### 3.1.1. Sentiment Analysis Using Social Media Data

Twitter is a social media platform on which users can post and interact with messages known as “tweets.” Registered users can post, like, and re-tweet the tweets from followed friends, while unregistered users can only see those that are in public status. Like the other social media platforms, Twitter has become a popular tool to connect people and transfer information and personal feelings. It also provides a convenient method to catch market sentiment. Due to its popularity, many investment communities have adopted the sentiment analysis from Twitter to predict the stock market trend. If Twitter users want to show that they share financial information, they can write a tweet including a stock ticker with a dollar sign. In this work, we performed sentiment analysis by analyzing each word in a tweet to obtain insights into users’ sentiment about a particular stock. The Natural Language Toolkit (NLTK) [47] contains various utilities that allowed us to manipulate and analyze linguistic data effectively. Among its advanced features are text classifiers; we can employ a pre-trained sentiment analyzer called valence aware dictionary and sentiment reasoner (VADER) to classify the tweet data into overall positive, neutral, and negative categories. The VADER library will return four values: (1) pos: the probability of the sentiment to be positive, (2) neu: the probability of sentiment to be neutral, (3) neg: the probability of the sentiment to be negative, and (4) compound: the normalized compound score, which calculates the sum of all lexicon ratings and takes values from −1 to 1. We classified the sentiment of the tweet into five classes (more positive, positive, more negative, negative, and neutral) by the following equation:

(1) $$Sentiment=\left\{\begin{array}{cc} more\;positive & if\;compound>0.5\\ positive & if\;compound\in \left[0.5,0\right)\\ neutral & if\;compound=0\\ negative & if\;compound\in \left(0,-0.5\right]\\ more\;negative & if\;compound<-0.5 \end{array}\right.$$

Some research on stock trends has shown that sometimes positive sentiment has an impact [48], while other times negative sentiment has an impact [49]. Therefore, it is important to track the sentiment feature because it may have different effects. We utilized a sentiment score to measure our tweet’s sentiment as the following equation:

(2) $$Sentiment\_score=\left\{\begin{array}{cc} 4 & if\;tweet\;is\;more\;positive\\ 3 & if\;tweet\;is\;positive\\ 2 & if\;tweet\;is\;neutral\\ 1 & if\;tweet\;is\;negative\\ 0 & if\;tweet\;is\;more\;negative \end{array}\right.$$

The overall sentiment score of a given day is the combined sentiment score of all the tweets for that particular day. As shown in Equation (2), if a particular day has a higher overall sentiment score, then it means that the sentiment positivity is higher on that day.

#### 3.1.2. Sentiment Networks

To evaluate the stock prediction performance using sentiment analysis, we utilized five machine learning methods in our studies such as random forest (RF), linear support vector classification (LinearSVC), Gaussian naive Bayes (GaussianNB), long short-term memory (LSTM), and 1D-CNN.

##### Random Forest (RF)

RF is an ensemble machine learning algorithm that is used to solve classification and regression problems. It has shown compelling efficiency for stock market prediction using sentiment analysis on media and news data [46]. A random forest algorithm involves constructing a large number of decision trees from bootstrap samples in a training dataset, like bagging. In general, a decision tree is a decision support technique that forms a tree-like structure. The decision-trees method combines the “bagging” idea and the random selection of features from training sets. A decision-tree method divides the training dataset into branches, which further segregates it into other branches. This process continues until reaching a leaf node. Unlike bagging, the processes of finding the root node and splitting the feature nodes will run randomly in a random forest method. We employed our random forest algorithm using skicit-learn [50].

Similar to [46], we set the min_samples_leaf equal to 1, and the criterion used Gini impurity. While the max_depth equaled 2 and the random_state parameter equaled 0, the other parameters received the default values.

##### Linear Support Vector Classification (LinearSVC)

Support vector machines (SVMs) are a set of supervised learning methods used for classification, regression, and outliers detection. The support vector machines method aims to find a hyperplane in an N-dimensional space that distinctly classifies the data points. The objective of SVM is to find a plane that has the maximum margin because maximizing the margin distance means that the future data points can be classified with more confidence. SVM has proved successful in the sentiment classification [27,33,41,46,51]. LinearSVC is a support vector machine that generates a linear classifier. We explored the LinearSVC in our work since LinearSVC has more flexibility in choosing penalties and the loss function, and it scales better to the larger number of samples.

In our problem settings, we set random_state equal to 42 and the class_weight parameter equal to “balanced,” while the other parameters received the default values. We also employed our LinearSVC algorithm using skicit-learn [50].

##### Gaussian Naive Bayes (GaussianNB)

A naive Bayes classifier is a supervised machine learning classification algorithm based on the Bayes theorem [52]. The algorithm is highly effective when the dimensionality of the inputs is high. Some researchers have applied naïve Bayes to classify text sentiment [27,51,53]. GaussianNB is a variant of naive Bayes that follows a Gaussian normal distribution and that supports continuous values. It is one of the most widely used machine learning algorithms. Similar to [46], we applied GaussianNB as one of the machine learning classifiers in our sentiment analysis problem. The Gaussian normal distribution is calculated using the following equation:

(3) $$f\left(x\right)=\frac{1}{\sqrt{2\pi }\sigma^{2}}\exp\left(-\frac{{\left(x-\mu \right)}^{2}}{2\sigma^{2}}\right)$$

where f(x), σ, and μ represent the probability density function, the standard deviation, and the mean, respectively. The Gaussian naive Bayes model calculates the means and standard deviation of input values for each class to summarize the distribution. At every data point, the z-score distance between that point and each class-mean is calculated and stored.

##### Long Short-Term Memory (LSTM)

**Figure 2:**
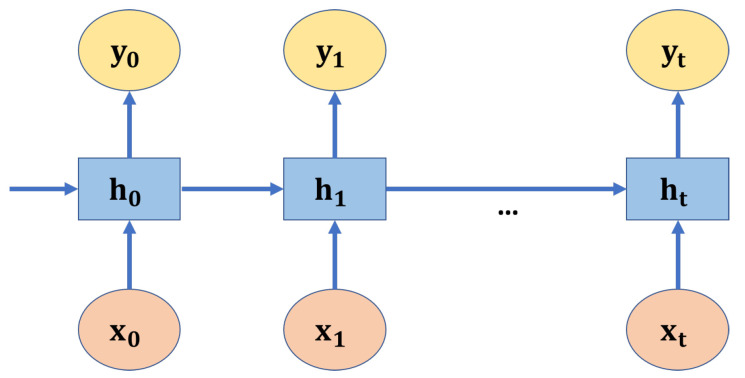
A recurrent neural network architecture.

A recurrent neural network (RNN) is a type of artificial neural network that maintains hidden internal states to model inputs with dependence through directed cyclic connections between its units. RNN is usually used to process sequential data or time-series data. Unlike traditional neural networks, the output of RNN depends on the primary elements within the sequence. For example, we wanted to predict the word following a given sentence. It is clear that the predicted word will have a semantic relationship with the other words in that sentence. RNN generates outputs based on current and previous inputs. The hidden states in RNN hold information on previous inputs. Figure 2 shows an RNN architecture unrolled into a full network, in which $x_{t}$, $h_{t}$, $y_{t}$ represents the input, hidden, and output state at the time *t*, respectively.

(4) $$h_{t}=\tanh\left(W_{xh}x_{t}+W_{hh}h_{t-1}+b_{h}\right),$$

(5) $$y_{t}=W_{hy}h_{t}+b_{Y},$$

where $W_{hh}$, $W_{hy}$, and $W_{xh}$ represent the hidden-to-hidden layer, the output-to-hidden layer, and the input-to-hidden layer weight matrices, respectively. $b_{h}$ and $b_{y}$ are the biases of the hidden and the output layers, respectively.

The LSTM network is an extension of RNN by adding three gates: a forget gate to control whether to forget the current state, an input gate to control if the input should be stored, an output gate to control whether to output the state. The activations of LSTM are calculated as the following equations:

(6) $$i_{t}=\sigma \left(W_{xi}x_{t}+W_{hi}h_{t-1}+W_{ci}c_{t-1}+b_{i}\right),$$

(7) $$f_{t}=\sigma \left(W_{xf}x_{t}+W_{hf}h_{t-1}+W_{cf}c_{t-1}+b_{f}\right),$$

(8) $$c_{t}=f_{t}c_{t-1}+i_{t}\tanh\left(W_{xc}x_{t}+W_{hc}h_{t-1}+b_{c}\right),$$

(9) $$o_{t}=\sigma \left(W_{xo}x_{t}+W_{ho}h_{t-1}+W_{co}c_{t}+b_{o}\right),$$

(10) $$c_{t}h_{t}=o_{t}\tanh\left(c_{t}\right),$$

where $i_{t}$, $f_{t}$, $c_{t}$, and $o_{t}$ denote the input gate, the forget gate, the cell activation vectors, and the output gate at time *t*. $b_{i}$, $b_{f}$, $b_{o}$, and $b_{c}$ are the biases of the gates.

LSTM has shown great success in solving sequence modeling problems such as machine translation, time series, and sentiment analysis [54,55,56]. In this work, we used a simple LSTM with three layers. The first LSTM layer contained thirty internal units followed by a dropout layer with a probability of 0.5. The second LSTM layer contained 256 units. The final dense layer with two units corresponded to the number of classes of our stock trend prediction.

##### 1D-CNN

**Figure 3:**
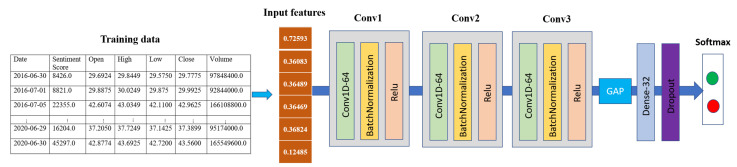
1D Convolutional neural network architecture for sentiment data.

In Wang’s work [57], it was demonstrated that a fully convolutional network (FCN) with global average pooling (GAP) achieves premium performance to other state-of-the-art approaches in time series data. Inspired by this work, we designed a 1D-CNN network for performing sentiment analysis for our stock price prediction. The selected parameters (the kernel size and the number of filters) were found via random search using Keras Tuner [58]. Our 1D-CNN sentiment network consisted of three 3 × 3 1D convolutional layers. Each layer comprised 64 channels, followed by the BatchNormalization layer and ReLu activation. There was a GAP layer with 32 units, ReLu activation, and one dropout layer with a dropout probability of 0.5. Finally, the softmax output layer with two units corresponded to the number of classes of our stock trend prediction. Figure 3 exhibits our 1D-CNN architecture.

### 3.2. CandleStickChart for Stock Trend Prediction

#### 3.2.1. Candlestick Chart

**Figure 4:**
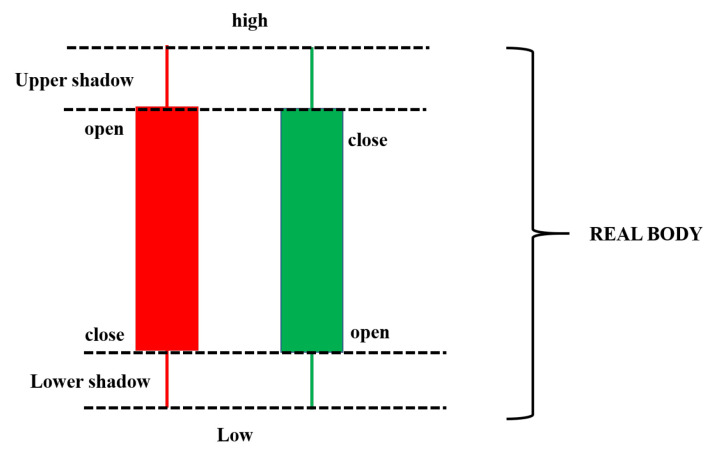
An example of a candlestick chart.

The candlestick chart was developed by Homma [59], a famous rice trader known in Sakata city, Japan. After that, many Japanese traders used it to predict the future prices in rice trading contracts [60]. A candlestick chart is a combination between a line-chart and a bar used to describe the price movements for a given period of time. Figure 4 shows an example of a candlestick chart. Candlestick charts consist of three components, such as an upper shadow, a lower shadow, and a real body. Each bar of the candlestick chart represents the information of a trading day, such as the open, the close, the low, and the high price. The thick part of the candlestick chart represents the different distances between the opening and closing prices. The different colors of the candlestick also indicate a different meaning. The real body will be filled in a red color if the open price is higher than the closing price. Otherwise, the real body will be filled with a green color. The upper and lower lines at the end of the real body represent the upper and lower shadows, respectively. The upper and lower shadows represents the high and low price ranges of candlestick charts in a given time period, respectively.

#### 3.2.2. Candlestick Chart Network

**Figure 5:**
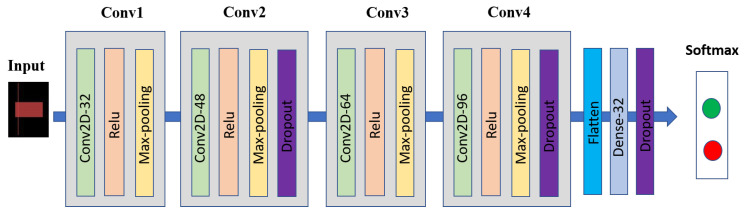
2D convolutional neural network architecture for candlestick chart data.

Our previous work [21] demonstrated that a 2D-convolutional neural network (2D-CNN) outperformed the other popular networks (VGG16, RestNet50, and random forest) for stock price movement prediction using candlestick chart data. Similar to our previous work [21], we employed this 2D-CNN for our candlestick chart network. The 2D-CNN architecture contained four 3 × 3 2D convolutional layers. Layer 1, layer 2, layer 3, and layer 4 had 32, 48, 64, and 96 channels, respectively. Each layer contained a 2 × 2 max pooling layer, with ReLu activation. There were three dropouts and a fully connected layer with 32 units, with a Relu activation. Finally, a soft-max output layer with two units represents stock trend prediction. Figure 5 exhibits our 2D-CNN architecture.

### 3.3. Joint Sentiment and Candlestick Chart Model

To improve our prediction result, we proposed a joint prediction model based on sentiment analysis and candlestick chart patterns that could incorporate all of a stock’s features. Our collaborative network included two branches: (1) the first branch was a 1D-CNN to perform sentiment analysis as described in Section 3.1.2. (2) The second branch performed image classification based on the candlestick charts through the 2D-CNN described in Section 3.2.2. Finally, the output of these branches was then concatenated, and they were fed into a standard set of dense layers where the last layer had two neurons to classify the respective stock price as increasing or decreasing in the near future. Figure 1 exhibits our joint prediction model.

### 3.4. Performance Measures

There were various statistics measures utilized to evaluate the performance of the classification model. In this study, we used the accuracy primary classification metric and three within-class classification metrics such as Precision, Recall, and the F-score.

The accuracy metric determines how good our model is, and it is a ratio of the number of correct predictions to the total observations. The accuracy metric is formulated as follows:

(11) $$Accuracy=\frac{number\;of\;correct\;predictions}{total\;number\;of\;predictions}=\frac{TP+TN}{TP+FP+TN+FN}$$

where TP is True Positive, representing that the increased future trend of the stock has been identified as an increasing trend; FP is False Positive, representing that the decreased future trend of the stock has been identified as an increasing trend; TN is True Negative, representing that the decreased future trend of stock has been identified as a decreasing trend; and FN is False Negative, representing that the increased future trend of stock has been identified as a decreasing trend.

The precision metric represents how precise our model is out of those predicted increased trends and how many of them are actual increasing trends. This metric is a good measure to determine when the cost of False Positive is high. The precision metric can be calculated as follows:

(12) $$Precision=\frac{TP}{TP+FP}$$

The recall metric calculates how many of the actual increasing trends our model captures by labeling it as an increasing trend (True Positive). This metric is a good measure to determine when the cost associated with False Negative is high. Recall that the metric can be calculated as follows:

(13) $$Recall=\frac{TP}{TP+FN}$$

The F-score was used to seek a balance between Precision and Recall. The value of the F-score was 1.0, indicating the perfect Precision and Recall. In contrast, the lowest value of the F-score was 0, indicating that either the Precision or the Recall was zero. The F-score is formulated as follows:

(14) $$F\text{-}score=2*\frac{Precision*Recall}{Precision+Recall}$$

Additionally, sometimes the accuracy metric values did not differ substantially between the models. Because stock traders are highly aware of the range of profits they can earn in stock trading, we proposed an accuracy_level metric to show the accuracy range between classification models. We defined the accuracy_level as the following equation:

(15) $$Accuracy\_level=\left\{\begin{array}{cc} 0, & accuracy\in \left[0,10\right)\%\\ 1, & accuracy\in \left[10,20\right)\%\\ 2, & accuracy\in \left[20,30\right)\%\\ 3, & accuracy\in \left[30,40\right)\%\\ 4, & accuracy\in \left[40,50\right)\%\\ 5, & accuracy\in \left[50,60\right)\%\\ 6, & accuracy\in \left[60,70\right)\%\\ 7, & accuracy\in \left[70,80\right)\%\\ 8, & accuracy\in \left[80,90\right)\%\\ 9, & accuracy\in \left[90,100\right)\%\\ 10, & accuracy=100\% \end{array}\right.$$

## 4. Experiment

In this section, we first describe the details of our data-collection and processing procedures. We then describe the improvement in sentiment analysis achieved by using a CNN. Finally, we demonstrate the improvement in our joint network by evaluating different aspects of the prediction of movement in stock prices and discuss the prediction of stock trends in different periods of time.

### 4.1. Data Collection and Processing

#### 4.1.1. Historical Data from Yahoo Finance

We selected five high-demand stocks in the United States: Apple, Tesla, IBM, Amazon, and Google (abbreviated as AAPL, TSLA, IBM, AMZN, and GOOG, respectively). Stocks were chosen based on a variety of factors, including their market capitalization in the top stock markets in the United States and their status as well-known stocks popular with global institutional investors. From Yahoo! Finance’s API, we collected historical time series data for each stock. The time period of stock data was four years of data from 30 June 2016 to 30 June 2020. We then extracted six features for each stock, including the date, the open, the high, the low, the close, and the volume corresponding to the stock’s traded date, the stock’s open price, the stock’s highest trading price, the stock’s lowest trading price, the stock’s close price, and the number of shares traded, respectively. The information of selected stocks is shown in Table 1.

In this work, we wanted to use features information in the current day as input $X_{i}$ to predict the future trend of stock in the next n days. It means that we wanted to find the difference between the closing price of a stock’s current day and the closing price after n days. The future trend’s label = 1 represents that the stock’s closing price will increase after n days. If the future trend’s label = 0, the stock’s closing price will decrease or not change after n days. The future trend’s label is determined with the following equation:

(16) $$Label=\left\{\begin{array}{cc} 1, & P_{t}>P_{t+n}\\ 0, & P_{t}\leq P_{t+n} \end{array}\right.$$

We would find the future trend of the stock price in the next 4 days, 6 days, 8 days, and 10 days. We randomly kept 20% of the data for testing and the rest of the data for training from our stock data.

#### 4.1.2. Sentiment Data from Social Media (Twitter)

To collect the tweets from Twitter for each particular stock, we utilized the snscrape [61] library, which allowed us to scrape tweets without the restrictions of Tweepy [62]. Snscrape is a scraper for social networking services (SNS). It will scrape things such as profiles, hashtags, or searches and returns the discovered items, e.g., the relevant posts. In our work, the Python application would obtain input parameters such as a search query, symbols of the selected stock, the start date, and the end date. All tweets of the selected stocks were downloaded in raw format between a start date and an end date. The tweet data contained four features: Date, TweetText, ReTweetCount, and LikeCount—which indicate the date on which the tweet was posted, the tweet’s content, the number of re-tweets, and the number of likes on that tweet, respectively. Table 2 shows an example of tweet data for AAPL stock.

We then processed the downloaded tweets by the following steps:

- Removing the unimportant/spam tweets that contained ReTweetCount and LikeCount values <5 and <10, respectively.
- Tweets were split and converted into word tokens.
- Removing the non-Ascii char, hyperlinks, punctuations, tokenize, stopwords(for example, the, are, is, an, etc.) inside the tweets because these components carry no useful information for sentiment analysis.

Finally, we applied the sentiment analysis for processed tweets on each day to obtain the overall sentiment score as described in Section 3.1 for that particular day. Table 3 shows an example of the final sentiment data format.

#### 4.1.3. From Historical Data to Candlestick Chart

**Figure 6:**
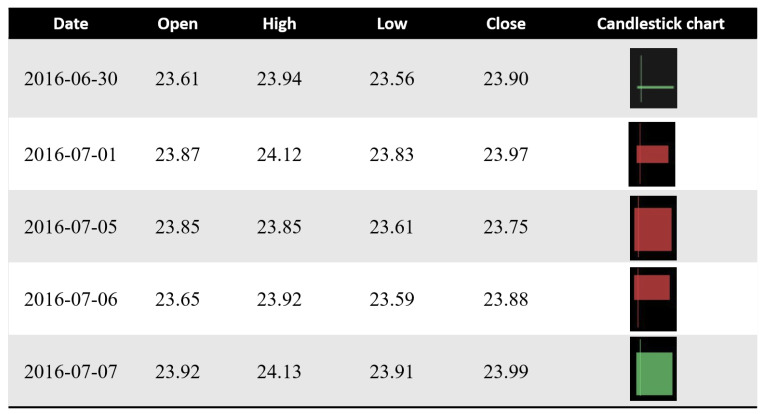
A view of candlestick chart data for stock price movement prediction.

A candlestick chart essentially has four features—the open, the high, the low, and the close—called OHLC. To create a candlestick chart, we utilized the mpl_finance module [37] containing a new matplotlib.finance API that makes it easier to generate financial plots. Figure 6 illustrates an example of candlestick data.

### 4.2. Experimental Setting

Deep learning models are full of hyperparameters; therefore, finding the best configuration in the CNN model is a time-consuming and resource-consuming process. In this work, we performed some procedures to determine the optimal values of hyperparameters to achieve the model with the highest performance. Those hyperparameters were the batch size, the optimizer function, and the learning rate. We applied a GridSearchCV approach from the scikit-learn wrapper class in Keras API to find the optimal batch size and optimizer function. We defined the grid search of batch size in the set of (8, 16, and 24) and the optimizer function in (Adam, SGD, and RMSprop). The GridSearchCV constructed and evaluated the model for each combination of these two set parameters using three-fold cross-validation.

To control the learning rate and save the best model, we applied ReduceLROnPlateau and Modelcheckpoint callbacks API in Keras. The learning rate would be reduced by a factor of 0.5 if there was no improvement after 20 continuous epochs. This process continued until reaching the lower bound. We set the coarse learning rate = 0.001 and the lower bound on the learning rate = 0.0001. Modelcheckpoint stored the best model according to the validation loss value at every epoch.

### 4.3. Improvement in Stock Trend Prediction Based on Sentiment Analysis and 1D-CNN

To choose an effective stock trend prediction model based on sentiment analysis, we conducted experiments using the five methods discussed in Section 3. We implemented each method to predict the stock trend for five high-demand stocks (AAPL, TSLA, IBM, AMZN, and GOOG) over various time periods (4 days, 6 days, 8 days, and 10 days). The highest accuracy is highlighted in bold. As shown in Table 4, The 1D-CNN model achieved more favorable performance than the random forest (RF), LinearSVC, GaussianNB, and LSTM models in most cases. We achieved the highest accuracy of 71.36% on AAPL stock for the prediction of the subsequent 8 days using the 1D-CNN model. By comparison, the model achieved the lowest accuracy of 46.73% for IBM stock in the prediction of the subsequent 4 days using RF classifiers. Therefore, we used this 1D-CNN for our sentiment analysis of stock trend prediction.

### 4.4. Improvement inStock Trend Prediction Based on the Joint Network

To further improve our stock prediction result, we proposed a collaborative network with multiple channels that can incorporate all the features from sentiment analysis and candlestick charts. In this section, we first evaluate different aspects of our collaborative network using accuracy evaluation and accuracy0level metrics on five high-demand stocks (AAPL, TSLA, IBM, AMZN, and GOOG). We then evaluate the stock trend prediction of our proposed model on different time periods.

#### 4.4.1. Ablation Study

In this section, we examine how each component in the proposed method affected our prediction performance. We evaluate three models: (1) sentiment analysis model, (2) candlestick chart model, and (3) our full model (joint network based on sentiment analysis and candlestick charts).

- Sentiment: the sentiment analysis network based on social-media data and the 1D-convolutional neural network described in Section 3.1.2.
- Candlestick chart: 2D-convolutional neural network based on candlestick-chart data described in Section 3.2.2.

Table 5 summarizes our ablation study results on different time periods (4 days, 6 days, 8 days, and 10 days). The highest accuracy and accuracy_level are highlighted in bold. We observed a significant performance improvement when the proposed method was used, indicating the benefits of stock prediction with a multichannel network. As shown in Table 5, rhe sentiment network seemed to work well with a 4-day time period, while the proposed method worked well for the other time periods (6 days, 8 days, and 10 days). The single-channel network using candlestick-chart data achieved the lowest results; however, it helped to improve the accuracy performance up by 8% when combing with the single-channel sentiment network in the multichannel proposed network.

The same conclusion with the evaluation using the accuracy_level metric; Table 5 shows that utilizing the multichannel proposed network achieved a more favorable outcome than a single sentiment network and single candlestick-chart network, indicating that stock traders have a higher chance of earning profit when using our proposed method.

#### 4.4.2. Stock Trend Prediction on Different Time Periods

**Figure 7:**
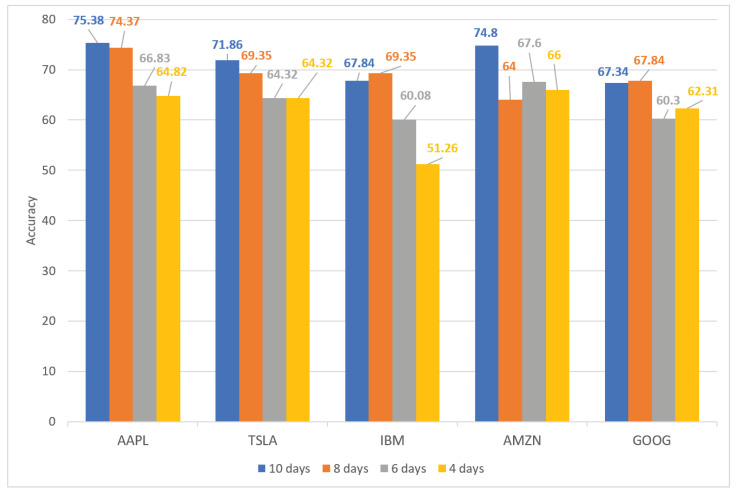
Performance of proposed method for five hot stocks (AAPL, TSLA, IBM, AMZN, and GOOG) on different period time (4, 6, 8, and 10 days).

Table 6 and Figure 7 show the performance of our proposed method for five high-demand stocks (AAPL, TSLA, IBM, AMZN, and GOOG) for different periods of time. The highest accuracy, precision, recall, and F-score of each stock are highlighted in bold. Figure 7 shows that the prediction accuracy increased gradually from day 4 to day 10. This indicates that stock performance over longer periods of time achieved a more favorable result than that over shorter periods of time. This may be because the stock’s movement trended over a longer period. Therefore, this contributes to the accuracy of predictions over longer periods of time. As listed in Table 6, the proposed method obtained the most favorable performance with 75.38% accuracy on AAPL stock over a 10-day time period. By contrast, IBM stock, over a time period of 4 days, achieved the least-favorable performance with 51.26% accuracy. Additionally, our model achieved the most balanced results for TSLA stock over a 10-day time period with 71.86% accuracy, 68.42% precision, 79.59% recall, and a 73.58% F-score.

## 5. Conclusions

In this study, we presented a new multichannel network for predicting stock prices based on five high-demand stocks (AAPL, TSLA, IBM, AMZN, and GOOG) selected by the size of their market capitalization. We first employed the Natural Language Toolkit to perform sentiment analysis for text content in Twitter tweets to obtain insights into users’ sentiments about a particular stock. From classifying sentiment analysis results, we obtained an overall sentiment score for a given day for the stock. We then generated candlestick chart images based on historical time series data by using computer graphic techniques to visualize the daily price and stock movement for a given stock. These two types of data were input into our joint multichannel network. Our joint network consisted of two branches: (1) the first branch included a 1D-CNN performing classifications based on sentiment analysis. (2) The second branch contained a 2D-CNN performing image classifications based on 2D candlestick chart images. The outputs of the two branches were concatenated and fed into a standard set of dense layers where the last layer computed the data and predicted the stock movement for the near future. The experiment results indicated that our proposed joint network achieved promising results for stock prediction and outperformed networks using either single sentiment data or candlestick charts alone. This indicates that using both types of data is more effective in the prediction of stock trends because both types of data can change and affect a stock’s price movement and traders’ decisions. The experiment results also indicated that the performance of stock prediction over longer periods of time achieved a more favorable result than did predictions made over shorter periods of time. Our proposed method achieved the most favorable performance with 75.38% accuracy for AAPL stock over a 10-day time period.

## Figures and Tables

**Table 1:** Selected stocks’ symbols, stock name, and stock exchange.

No	Stock Name	Ticker Symbol	Country/Stock Exchange
1	Apple	AAPL	USA
2	Tesla	TSLA	USA
3	IBM	IBM	USA
4	Amazon	AMZN	USA
5	Alphabet (Google)	GOOG	USA

**Table 2:** A view of tweet data for stock price movement prediction.

Date	TweetText	RetweetCount	LikeCount
30 June 2020	Jim Cramer break favorite stock…	7	72
30 June 2020	Today Recap look ahead spx aapl…	3	37
30 June 2020	top 10 holding stock portfolio…	0	10
30 June 2020	Setting team success Look name…	3	11
30 June 2020	MNST COO AAPL…	0	56
30 June 2020	top 10 holding make 62 portfol…	5	74
30 June 2020	SPOT AAPL ht @a16z…	3	16

**Table 3:** A view of sentiment data for stock price movement prediction.

Date	SentimentScore	Open	High	Low	Close	Volume
30 June 2016	36	23.61	23.94	23.56	23.90	143,345,600
1 July 2016	86	23.87	24.12	23.83	23.97	104,106,000
5 July 2016	182	23.85	23.85	23.61	23.75	110,820,800
6 July 2016	218	23.65	23.92	23.59	23.88	123,796,400
7 July 2016	240	23.92	24.13	23.91	23.99	100,558,400
8 July 2016	262	24.12	24.22	24.01	24.17	115,648,400
11 July 2016	322	24.19	24.41	24.18	24.25	95,179,600
12 July 2016	346	24.29	24.42	24.28	24.35	96,670,000

**Table 4:** Accuracy improvement of stock trend prediction based on sentiment analysis and 1D-CNN.

Stock	Time Period	RF	LinearSVC	GaussianNB	LSTM	1D-CNN
AAPL	10 days	62.81%	51.76%	58.79%	62.81%	**71.36%**
	8 days	61.31%	56.28%	60.30%	60.30%	**71.86%**
	6 days	56.78%	55.28%	59.30%	57.29%	**65.33%**
	4 days	61.31%	51.76%	55.28%	60.80%	**67.84%**
TSLA	10 days	60.80%	55.78%	58.29%	**64.82%**	63.32%
	8 days	57.29%	55.78%	56.28%	60.30%	**63.32%**
	6 days	58.79%	54.77%	52.76%	61.81%	**68.34%**
	4 days	57.29%	55.78%	57.29%	54.27%	**59.80%**
IBM	10 days	60.30%	62.81%	61.31%	60.80%	**64.82%**
	8 days	59.80%	60.30%	59.80%	62.31%	**64.82%**
	6 days	55.28%	56.28%	59.30%	56.78%	**59.80%**
	4 days	46.73%	56.78%	54.27%	**57.79%**	52.76%
AMZN	10 days	64.80%	52.80%	61.20%	62.80%	**69.60%**
	8 days	61.60%	53.60%	60.00%	58.00%	**64.80%**
	6 days	61.20%	58.40%	59.60%	56.40%	**61.60%**
	4 days	**64.40%**	60.80%	64.00%	60.40%	61.60%
GOOG	10 days	55.28%	59.30%	53.77%	55.78%	**67.34%**
	8 days	57.29%	59.30%	56.28%	56.28%	**67.34%**
	6 days	56.78%	57.29%	57.29%	**58.79%**	**58.79%**
	4 days	54.27%	53.77%	56.78%	59.30%	**62.81%**

**Table 5:** Accuracy improvement in stock trend prediction based on the joint network.

Stock	Period Time	Sentiment	Candlestick Chart	Joint Network
		Accuracy (Accuracy_Level)	Accuracy (Accuracy_Level)	Accuracy (Accuracy_Level)
AAPL	10 days	71.36% (**7**)	62.81% (6)	**75.38% (7)**
	8 days	71.86% (**7**)	61.31% (6)	**74.37% (7)**
	6 days	65.33% **(6)**	57.29% (5)	**66.83% (6)**
	4 days	**67.84% (6)**	61.31% **(6)**	64.82% **(6)**
TSLA	10 days	63.32% (6)	50.75% (5)	**71.86% (7)**
	8 days	63.32% **(6)**	52.76% (5)	**69.35% (6)**
	6 days	**68.34% (6)**	56.28% (5)	64.32% **(6)**
	4 days	59.80% (5)	52.76% (5)	**64.32% (6)**
IBM	10 days	64.82% **(6)**	47.74% (4)	**67.84% (6)**
	8 days	64.82% **(6)**	49.25% (4)	**69.35% (6)**
	6 days	59.80% (5)	49.25% (4)	**60.08% (6)**
	4 days	**52.76% (5)**	44.72% (4)	51.26% **(5)**
AMZN	10 days	69.60% (6)	61.20% (6)	**74.80% (7)**
	8 days	**64.80% (6)**	57.60% (5)	64.00% **(6)**
	6 days	61.60% **(6)**	56.40% (5)	**67.60% (6)**
	4 days	61.60% **(6)**	60.40% **(6)**	**66.00% (6)**
GOOG	10 days	**67.34% (6)**	54.77% (5)	**67.34% (6)**
	8 days	67.34% **(6)**	56.28% (5)	**67.84% (6)**
	6 days	58.79% (5)	56.78% (5)	**60.30% (6)**
	4 days	**62.81% (6)**	57.29% (5)	62.31% **(6)**

**Table 6:** Performance of proposed method for five hot stocks (AAPL, TSLA, IBM, AMZN, and GOOG) on different time periods (4, 6, 8, and 10 days).

Stock	Time Period	Accuracy	Precision	Recall	F-Score
AAPL	10 days	**75.38%**	**68.66%**	**62.16%**	**65.25%**
	8 days	74.37%	76.00%	49.35%	59.84%
	6 days	66.83%	64.18%	50.59%	56.58%
	4 days	64.48%	65.22%	19.48%	30.00%
TSLA	10 days	**71.86%**	**68.42%**	**79.59%**	**73.58%**
	8 days	69.35%	70.37%	60.64%	65.14%
	6 days	64.32%	60.53%	52.87%	56.44%
	4 days	64.32%	60.75%	69.15%	64.68%
IBM	10 days	67.84%	**72.73%**	61.53%	66.67%
	8 days	**69.35%**	70.83%	**67.33%**	**69.04%**
	6 days	60.80%	65.33%	48.51%	55.68%
	4 days	51.26%	57.83%	43.64%	49.74%
AMZN	10 days	**74.80%**	68.09%	**65.98%**	**67.02%**
	8 days	64.00%	61.11%	41.51%	49.44%
	6 days	67.60%	**68.42%**	47.71%	56.22%
	4 days	66.00%	60.94%	39.39%	47.85%
GOOG	10 days	67.34%	**82.05%**	35.56%	49.61%
	8 days	**67.84%**	78.05%	**36.78%**	**50.00%**
	6 days	60.30%	57.78%	30.23%	39.69%
	4 days	62.31%	66.67%	23.53%	34.78%
